# Geochemical evidence for a widespread mantle re-enrichment 3.2 billion years ago: implications for global-scale plate tectonics

**DOI:** 10.1038/s41598-020-66324-y

**Published:** 2020-06-11

**Authors:** Hamed Gamal El Dien, Luc S. Doucet, J. Brendan Murphy, Zheng-Xiang Li

**Affiliations:** 1 Earth Dynamics Research Group, The Institute for Geoscience Research (TIGeR), School of Earth and Planetary Sciences, Curtin University, GPO Box U1987, Perth, WA 6845 Australia; 2 Geology Department, Faculty of Science, Tanta University, 31527 Tanta, Egypt; 3 Department of Earth Sciences, St. Francis Xavier University, Antigonish, Nova Scotia Canada

**Keywords:** Geochemistry, Geodynamics, Petrology

## Abstract

Progressive mantle melting during the Earth’s earliest evolution led to the formation of a depleted mantle and a continental crust enriched in highly incompatible elements. Re-enrichment of Earth’s mantle can occur when continental crustal materials begin to founder into the mantle by either subduction or, to a lesser degree, by delamination processes, profoundly affecting the mantle’s trace element and volatile compositions. Deciphering when mantle re-enrichment/refertilization became a global-scale process would reveal the onset of efficient mass transfer of crust to the mantle and potentially when plate tectonic processes became operative on a global-scale. Here we document the onset of mantle re-enrichment/refertilization by comparing the abundances of petrogenetically significant isotopic values and key ratios of highly incompatible elements compared to lithophile elements in Archean to Early-Proterozoic mantle-derived melts (i.e., basalts and komatiites). Basalts and komatiites both record a rapid-change in mantle chemistry around 3.2 billion years ago (Ga) signifying a fundamental change in Earth geodynamics. This rapid-change is recorded in Nd isotopes and in key trace element ratios that reflect a fundamental shift in the balance between fluid-mobile and incompatible elements (i.e., Ba/La, Ba/Nb, U/Nb, Pb/Nd and Pb/Ce) in basaltic and komatiitic rocks. These geochemical proxies display a significant increase in magnitude and variability after ~3.2 Ga. We hypothesize that rapid increases in mantle heterogeneity indicate the recycling of supracrustal materials back into Earth’s mantle via subduction. Our new observations thus point to a ≥ 3.2 Ga onset of global subduction processes via plate tectonics.

## Introduction

Although plate tectonics is now well accepted as the paradigm for Earth’s evolution in the Phanerozoic eon, the question of when these processes began is still controversial^1^. Estimates are based mainly on crustal observations, and range from early Archean to the late Neoproterozoic^1^. Resolution of this debate is fundamental to our understanding of the evolution of Earth systems. A key process of plate tectonics is widespread subduction^2,3^. Subduction zones recycle terrestrial materials back into Earth’s mantle as the subducting slab sinks and re-equilibrates within Earth’s interior^2,4,5^. Fluids and magmas released from sediments and crustal materials in the vicinity of the subducting slab facilitate melting of the upper mantle wedge creating arc basalts with specific trace element signatures (such as elevated large ion lithophile elements (LILE): Ba, Pb, U, Sr, As, B, and Cs)^6,7^ that reflect enrichment of the sub-arc mantle source (compared to mid-ocean ridge basalts^8,9^). Some of these recycled terrestrial materials also invade the deeper mantle^10^, causing enrichment of the deep mantle in LILE and light rare earth elements (LREE) and promoting a geochemical and isotopic heterogeneity that characterizes basalts derived from mantle plumes^9,11–13^. It is commonly believed that before the plate tectonics regime, a chemically stratified Earth had a relatively homogeneous mantle composition^14,15^ (due to the lack of large/global-scale recycling of terrestrial materials into the upper and lower mantle) that was depleted in highly incompatible elements (e.g. Ba, Pb, Rb, Cs, Sr, and U) but enriched in high field strength elements (e.g. Nb and Ta)^16^. So, a globally-detectable large change in the mantle heterogeneity^9,12,17^, caused by a refertilization/re-enrichment in incompatible and fluid mobile elements, and a step-change in Nd isotope systematics of the upper and lower mantle-derived materials, could identify the onset of global-scale subduction and plate tectonic processes. Thus, tracking the isotopic and chemical heterogeneities of the Earth’s upper and lower mantle through the Archean and Early Proterozoic may provide a new way of identifying when plate tectonics started.

### Geochemical tracer for crustal recycling

Most previous estimates of when plate tectonics commenced were based on proxies recorded in continental crustal rocks^18–22^ which are only indirectly related to mantle processes, and may intrinsically have a preservation bias and/or reflect regional rather than global processes^18,23–29^. In order to identify when widespread global-scale mantle refertilization/re-enrichment occurred, we investigate the composition of the mantle directly by examining the global database of Archean-Early Proterozoic mafic-ultramafic rocks focusing on their Sm-Nd isotopic systematics and on petrogenetically-sensitive trace element ratios.

Samarium and Neodymium have very similar chemical behaviour (i.e., similar ionic radii and the same valency). As the Sm/Nd ratio is robust to the effects of alteration and metamorphism and is not significantly affected by crystal fractionation, this ratio typically reflects source composition^11,30–32^. As the depleted mantle reservoir retains Sm over Nd, its Sm/Nd ratio (~ 0.5) is greater than the bulk earth chondritic (~ 0.32) and typical continental crust (~ 0.2) values^31,32^. Thus, ^147^Sm to ^143^Nd decay over geological time would yield a significantly higher ^143^Nd/^144^Nd ratio in magmas derived from depleted mantle compared to contemporary magmas derived from a crustal reservoir^31^. As magmas passively acquire the ^143^Nd/^144^Nd initial ratio of their source^31^, differences in ɛNd (the relative deviation of the ^143^Nd/^144^Nd initial ratio from the chondritic value, ɛNd = 0) in mafic/ultramafic rocks constrain the evolution of the mantle source. Over geological time, the depleted mantle isotopically evolves toward more positive ɛNd values but the crust evolves towards negative values^31,32^. Thus, shifting of ɛNd of mafic-ultramafic rocks to less positive values identifies when a significant contribution of terrestrial materials to the mantle source occurred^11,12,30,31^.

In addition, the ratios of incompatible fluid-mobile elements (FMEs: Ba, Pb, Rb, Sr, and U) to relatively immobile elements such as high field strength elements (HFSEs: Nb and Ta) and rare earth elements (REE) are excellent tracers for the invasion of fluids and magmas derived from the recycling of sediments (such as Ba/La and Ba/Nb)^33–35^ and of continental crust materials (such as U/Nb)^9,12^ into mantle sources^8,36^. FMEs are transferred to the crust during subduction dehydration and arc magmatism^6,37^, but HFSEs are retained in the mantle source by minerals such as amphibole and rutile^38^. Such trace element pairs/ratios, with similar incompatibility but with very distinct chemical behaviours, are particularly useful because they are insensitive to alteration/metamorphism, and are less fractionated during partial melting^9,12,15^. Thus, tracking ratios such as Ba/La, Ba/Nb, U/Nb, Pb/Nd and Pb/Ce in addition to Nd isotopes in mafic and ultramafic magmatic products during the Archean and Proterozoic eons could precisely identify the time when heterogeneities in their respective upper and lower mantle sources originated, as well as source chemistry differences and the change of mantle trace element budget.

## Results

**Figure 1:**
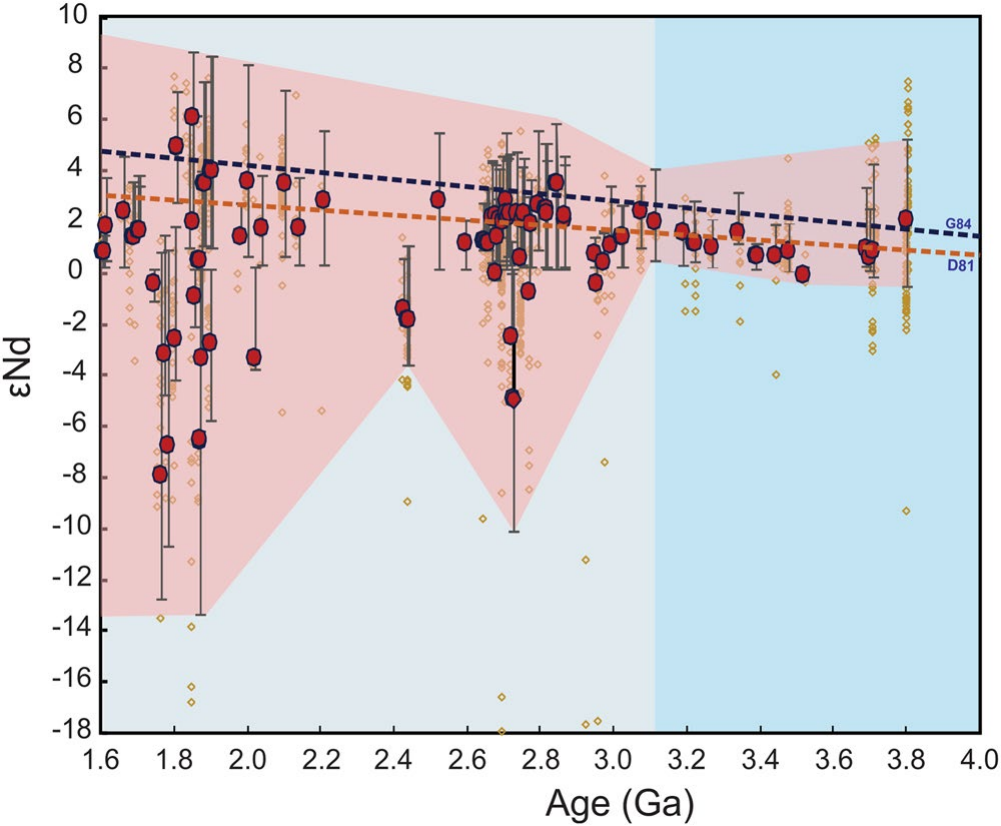
^143^Nd/^144^Nd ratio (represented as εNd) vs. age plot for Archaean and Proterozoic basaltic rocks and komatiites (data from Spencer *et al*.^39^**)**. Brown circles represent individual samples. Red dots represent the median of samples with the same age, and the associated error bars spans across the middle 50% of the data, called the median data range here. The red field represents the envelope for the median range. The large variation in the mean εNd values after ~3.2–3.0 Ga suggests an isotopic shift in the mantle source of the basaltic rocks and the komatiites. The depleted mantle curves are shown for comparison^70,71^.

**Figure 2:**
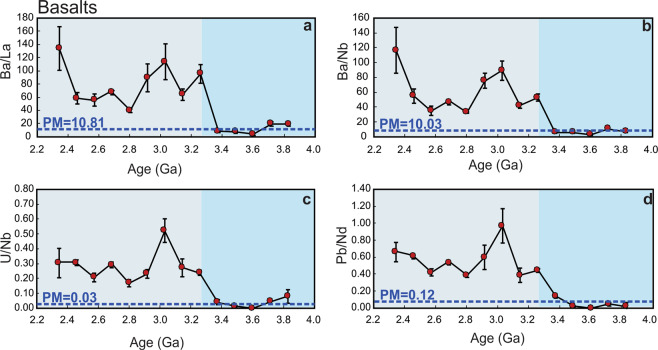
Time evolution of fluid-mobile-elements/immobile-elements in the basaltic rock datasets. Ba/La (**a**), Ba/Nb (**b**), U/Nb (**c**), and Pb/Nd (**d**). All ratios show an abrupt increase at ~3.25 Ga. Dotted horizontal lines are the primitive mantle values (PM; Ba/La = 10.81, Ba/Nb = 10.03, U/Nb = 0.03, Pb/Nd = 0.12)^47^. Error bars in a–d show the 2-standard errors of the means.

**Figure 3:**
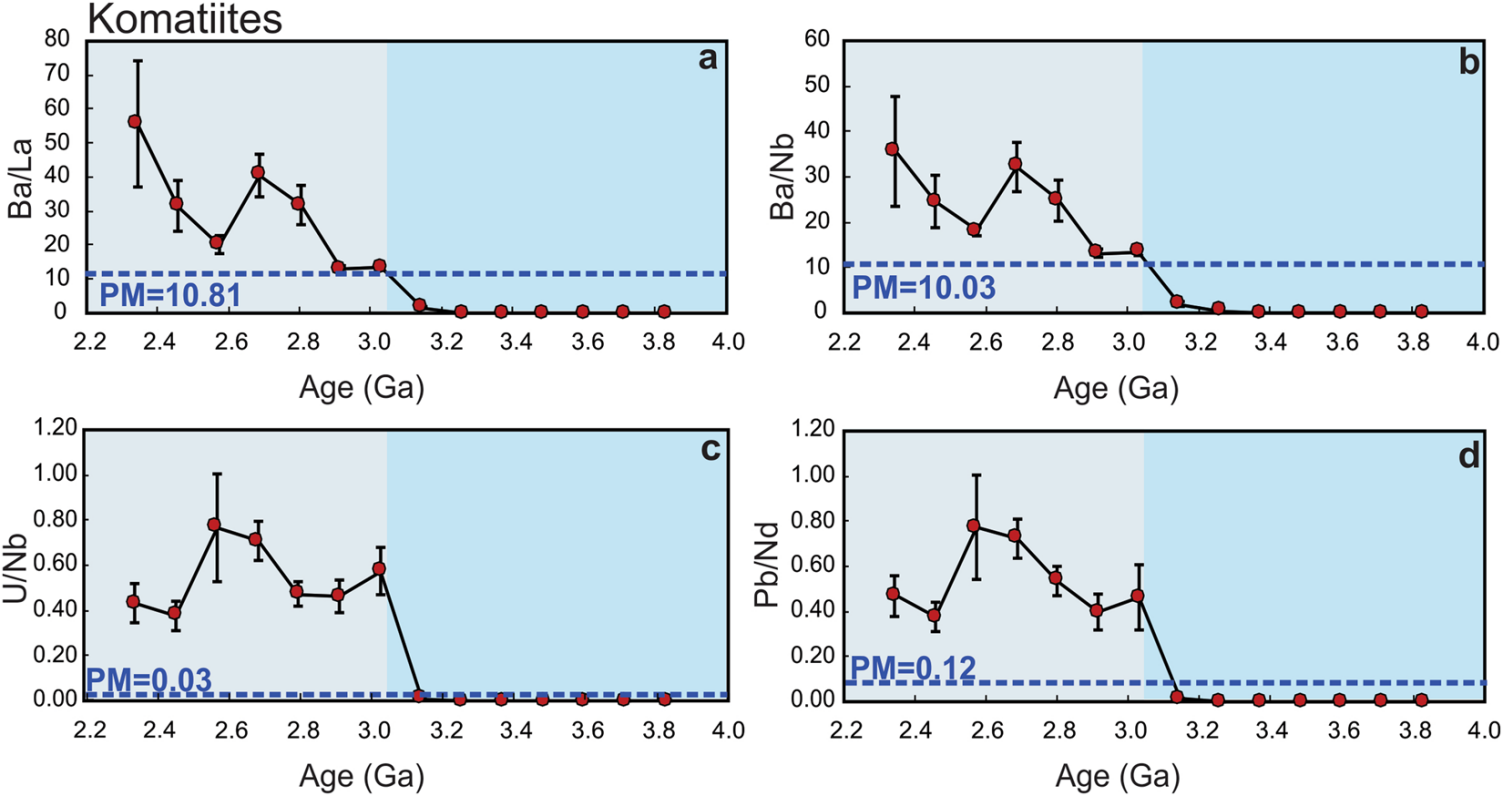
Time evolution of fluid-mobile-elements/immobile-elements in the komatiite datasets. Ba/La (**a**), Ba/Nb (**b**), U/Nb (**c**), and Pb/Nd (**d**). All ratios show an abrupt increase at ~3.15 Ga. Dotted horizontal lines are the primitive mantle values (PM; Ba/La = 10.81, Ba/Nb = 10.03, U/Nb = 0.03, Pb/Nd = 0.12)^47^. Error bars show 2-standard errors of the means.

We compiled a database consisting of major and trace element whole-rock and Nd isotopes^39^ of ~6,250 analyses from mafic and ultramafic rocks with reliable crystallization age and geospatial location for each sample (see methods). The studied samples are widely representative of all the continents and cratons, and span the Archean–Early Proterozoic time range (3.8–2.2 Ga) (Supplementary Figures 1–4 and Supplementary Tables 1 and 2). The database includes primary mantle melts represented by basaltic rocks and komatiites. Using the variation of the means of Nd isotopes (as ɛNd) in samples of the same age, and a statistical bootstrapping method^40^ on the basaltic and komatiitic rocks focusing on Ba/La, Ba/Nb, U/Nb, Pb/Nd and Pb/Ce ratios, we identify a significant change in mantle geochemical composition after ~3.25 Ga for basaltic rocks and after ~3.15 Ga for komatiites (Figs. 1–3 and Supplementary Figure 5).

Figure 1 displays a significant shift in the range of εNd values of basaltic rocks and komatiites that span the Paleo- to Meso-Archean transition (~3.2 Ga). Although specific suites may show a range in values implying the shift may occur locally at earlier times, these shifts are not recognizable when viewed from the perspective of the global database, indicating they are probably local in scale. For example, Nd isotopic data of the Greenland Eoarchean (3.8–3.7 Ga) basaltic rocks extend to negative εNd (attributed to mantle contamination^41,42^) although on average, the data from that time interval plot at positive εNd^43–46^ More generally, the averages of εNd in basaltic and komatiitic suites before ~3.2 Ga show little variation, ranging between +0.02 and +2.2. After ~3.2 Ga, however, εNd averages for basaltic and komatiitic rocks show an abrupt decrease from −1.5 (at ~3.0 Ga) to −8.0 (at ~1.7 Ga), which we interpret to reflect the onset of global-scale heterogeneity in their upper and lower (respectively) mantle sources (Fig. 1). This analysis provides robust evidence for global-scale, pene-contemporaneous contamination/refertilization of the mantle source for both mafic and ultramafic rocks, beginning after ~ 3.2 Ga in the upper mantle (inferred from basaltic rocks) but also affecting the lower mantle (inferred by komatiites) as the influx of LREE-enriched crustal materials yield negative εNd values^11,31^.

Trace element ratios (i.e., Ba/La, Ba/Nb, U/Nb, Pb/Nd and Pb/Ce) of basalts and komatiites are widely accepted to track the recycling of terrestrial materials into mantle sources^9,12^. Figures 2 and 3 monitor the best estimate of the average composition of these trace element ratios through time, and are reported as means with associated 2-standard-error (95% confidence interval) uncertainties of intervals between 2.2 and 4.0 Ga. These ratios display a systematic increase in both magnitude and variability, mainly after ~3.2 Ga. In general, Figure 2 and supplementary Figure 5a both display abrupt increases in the moving means of Ba/La, Ba/Nb, U/Nb, Pb/Nd and Pb/Ce ratios in basaltic rocks after ~3.25 Ga. In addition, the average mean values of all ratios of komatiites after ~3.15 Ga are highly enriched compared to komatiites older than ~ 3.15 Ga (Fig. 3 and Supplementary Figure 5b).

There is some evidence supporting localized subduction in specific suites before ca. 3.2 Ga. For example at 3.8–3.7 Ga, some ratios such as Ba/La (20.15–19.43) and U/Nb (0.08–0.05) are somewhat higher than estimates of equivalent ratios in the primitive mantle (PM)^47^ (Ba/La = 10.81 and U/Nb = 0.03, respectively (Fig. 2a,c). These higher values may reflect a subduction zone-like signature such as that proposed for the Isua greenstone belt, SW Greenland according to field and geochemical data interpretations^24,26,48–50^. Also, at 3.4–3.3 Ga, U/Nb (0.04) and Pb/Nd (0.14) are slightly higher than PM values (0.03 and 0.12, respectively)^47^ (Fig. 2c,d) which may reflect crustal contamination of the mantle source for the Barberton greenstone belt, the Kaapvaal craton^28,51–53^. However, when viewed from the perspective of the global database, these ratios become statistically detectable only around 3.25 Ga (e.g. Ba/La = 96.31, Ba/Nb = 53.32, U/Nb = 0.24 and Pb/Nd = 0.45 compared to the PM-like values and those before 3.25 Ga (Fig. 2). From this perspective, contamination of the mantle by LILE- and LREE-enriched recycled continental materials during subduction would have been localized and relatively minor in the Palaeoarchaean and the Eoarchaean, but became a global process at ca. 3.2 Ga. The observed time lag (~100 Ma) in the increase in those ratios between basalts and komatiites could reflect the transit time of subducted slabs from upper mantle (contaminated basaltic rocks) to the lower mantle (contaminated komatiites). Taken together, these results suggest a fundamental and global change in the upper and lower mantle source composition of basalts and komatiites through refertilization/replenishment of fluid-mobile elements at the start of the Mesoarchean, which is consistent with the negative εNd values after ~3.2–3.0 Ga (Fig. 1).

## Discussion

Our observed abrupt changes could be attributed to either (1) crustal contamination of ascending mantle-derived magma^54^ or (2) contamination of the mantle source by either subduction or delamination^14,54,55^. Crustal contamination/mixing of mafic and ultramafic magmas during their emplacement can be evaluated using the Th/Yb ratio, which is widely accepted as a powerful tracer of this process^56^. Supplementary Figures 6 and 7 show the covariation between the studied ratios and Th/Yb in both basaltic rocks and komatiites before and after 3.25 Ga and 3.15 Ga, respectively, compared with such ratios in Archean continental crust (i.e., tonalite-trondhjemite-granodiorite (TTGs) dataset)^57^. These plots show that Th/Yb has no relationship with Ba/La, Ba/Nb, U/Nb, Pb/Nd and Pb/Ce in either basaltic rocks (Supplementary Figure 6) or komatiites (Supplementary Figure 7). In addition, the Th/Yb values are very low compared to those typical of TTGs, providing clear evidence that no significant contamination of basaltic or komatiitic rocks by Archean crust occurred during their emplacement. Moreover, Archean basaltic and komatiitic rocks plot on an array that is parallel to the oceanic mantle array (MORB-OIB array) on the Th/Yb vs. Nb/Yb diagram (Supplementary Figure 8)^56^. This trend is similar to that of a modern-arc array, suggesting derivation of those rocks from a metasomatized/re-enriched mantle source^56^, and contrasts with the oblique trend displayed by TTGs (Supplementary Figure 8). The similarly low Th/Yb in post-3.2 Ga komatiites and basalts compares favourably with modern-arc basalts (Supplementary Figure 8), suggesting that the enrichment in these ratios was source-dependent and the mantle inherited these features before the generation of these mafic and ultramafic magmas.

The similarity between the average mean values of the studied trace element ratios in basalts and komatiites before ~3.25–3.15 Ga with primitive mantle estimates (PM)^47^ (Figs. 2, 3 and Supplementary Figures 5, 8) indicates the existence of a primitive-like and/or quasi-homogeneous mantle with only minor and local terrestrial inputs before ~3.25–3.15 Ga. In contrast, the average mean values of the studied ratios of basalts and komatiites after ~3.25–3.15 Ga are highly enriched compared to the primitive mantle (Figs. 2, 3 and Supplementary Figures 5, 8). Such an interpreted abrupt change in mantle composition is also reflected in the source of TTGs, generally considered to be juvenile crust newly extracted from the upper mantle^57^. As shown in Supplementary Figure 9, the same ratios (except for U/Nb) in TTGs not only have the same contents as their parent mafic rocks (Supplementary Figure 6) but also show the same abrupt change after ~3.3–3.2 Ga^57^. Similarly, a recent study on Jack Hills zircons (4.3–3.3 Ga) suggests a small yet notable change in Earth’s crustal composition between the Hadean and the Mesoarchean^58^.

Mantle refertilization/re-enrichment at ~3.2 Ga could have occurred by two major processes — sagduction/delamination^55^ or subduction^54^. Sagduction/delamination of dense residue after TTG formation would have facilitated the refertilization of the upper mantle with crust-like chemical and isotopic signatures^14,55^, but this process would have had less influence on the composition of the lower mantle^15,55^. Therefore, a sagduction/delamination scenario is not consistent with our analysis of komatiites (Fig. 3) which are thought to have been derived from the lower mantle, and represent the products of mantle plumes^17^. Komatiites younger than ~3.15 Ga are enriched in petrogenetically-indicating trace element ratios suggesting a re-enriched/metasomatized mantle source. Consistent with trace element results, εNd values of komatiites and basaltic rocks both show a pronounced abrupt-shift to lower values at ~3.0 Ga (Fig. 1). This shift is powerful evidence that recycling of subducted sediments and crust affected the composition of both the upper mantle and the deep mantle plume source on a global scale^11,12,30^, indicating that subduction into the lower mantle was widely/globally operative by ~3.2 Ga. These results are consistent with oxygen and hydrogen isotopes studies of the 3.2 Ga Barberton komatiites, South Africa that suggest mantle source heterogeneity by then^28,59^.

Some previous work^18,19,23^, though based on more indirect measures of global mantle composition than we present here, support our first-order conclusions. Shirey and Richardson^23^ interpret the appearance at ca. 3.2 Ga of eclogitic inclusions in diamonds from kimberlite pipes of the Kaapvaal craton to require subduction processes. Analysis of Hf-O zircon data in crustal rocks, which tracks the recycling of supracrustal materials, provides evidence for a step change at ca. 3 Ga indicative of the onset of subduction^18,19^. Recently, Sobolev and Brown^3^ hypothesize that the evolution and start of plate tectonics on Earth were facilitated by accumulation of sediments at the continental edges and trenches, which acted as a lubricant for the emergence and stabilization of subduction processes since the Mesoarchean (3.2–2.8 Ga). Our new observations, based on direct products of mantle melting, identify geochemical tracers of sediments recycling into both the upper and lower mantle (i.e., Ba/La and Ba/Nb) in both basaltic and komatiitic rocks, show that the abrupt increase occurred at ~3.2 Ga (Figs. 2 and 3). In addition, Gamal El Dien *et al*.^60^, using Mg, Ni and Cr elements in basaltic rocks show a consistent and rapid drop at ~3.2–3.0 Ga that indicates an abrupt change in mantle potential temperature at the start of global-scale plate tectonics. Although we cannot rule out the presence of intermittent stagnant lid tectonics along with plate tectonics after ~3.2 Ga^61^, our analysis suggests mass transfer from the surface to the deep mantle from ~3.2 Ga, a process most feasibly accomplished through subduction and plate tectonics.

Our interpretation assumes no dramatic continental crustal growth at around 3.2 Ga^62–64^. However, if there was a spike of global continental crustal growth at ca. 3.2 Ga (as argued by some^19,65,66^), then global plate tectonics could have started earlier than 3.2 Ga but mantle re-enrichment may not be as pronounced due to the relatively small amount of continental crust^67–69^.

Overall, our work points to a profound mantle re-enrichment event at ca. 3.2 billion years ago, interpreted to indicate the start of global-scale plate tectonics no later than that time.

## Methods

We compiled a database of basaltic rocks (n = 3,127) and komatiites (n = 2,740) for major and trace elements (including rare earth elements) mainly using the Georoc repository (Supplementary Data 1, 2). We cross-checked every sample with their original reference to verify its magmatic age and location (continent, craton and formation). Samples with no age constraints were excluded. All the selected samples have age estimates and age error less than ±100 Myr, sample ID and geospatial sample locations. The basaltic and komatiitic rocks in the selected database range in age of 3.8–2.4 Ga and 3.8–2.0 Ga, respectively. The basaltic rock database is composed mainly of basalts and basaltic andesites with 40–55 wt % SiO_2_, MgO <12 wt% and total alkali (K_2_O + Na_2_O) <5 wt %. To obtain an optimal distribution estimate of trace element ratios for mantle-derived melts (basalts and komatiites) and minimize sampling and preservation bias, we performed a weighted bootstrap resampling of the selected database following the method of Keller and Schoene^40^ using the Matlab MIT open-source code, available at https://github.com/brenhinkeller/StatisticalGeochemistry. All the fluid-mobile-elements/immobile-elements plots for basalts and komatiites in this paper were made using bootstrap-resampled data.

Nd isotopes database of Archaean and Proterozoic basaltic rocks and komatiites were taken from Spencer *et al*.^39^. Nd data were filtered, and only analyses with magmatic age constraints better than ±100 Myr were used. Only Georoc analyses that included ^143^Nd/^144^Nd along with Sm and Nd concentrations were used. The ^147^Sm/^144^Nd ratio was determined using the atomic weights and abundances with the following equation:

$${}^{147}Sm{/}^{143}Nd=\frac{Sm\,ppm}{Nd\,ppm}\ast \frac{Abs{.}^{147}Sm\ast At.wt.Nd}{Abs{.}^{144}Nd\ast At.wt.Sm}$$

The tonalite-trondhjemite-granodiorite rock database (TTG; sample number = 1,230) was collected from Johnson *et al*.^57^ The change in the median data range before and after 3.3–3.2 Ga is highlighted by rectangular shades of different colours (Supplementary Figure 9). Also, the average of medians for data within each rectangular shade is shown with horizontal bar. As many samples do not contain all the ratios used, the density of the data differs between plots.

## Supplementary information

Supplementary Information.

Supplementary Information2.

Supplementary Information3.

## Data Availability

All the data that are necessary for evaluating the findings of this study are available within this article and it’s Supplementary Information
