# Supplementary material for: Geochemical evidence for a widespread mantle re-enrichment 3.2 billion years ago: implications for global-scale plate tectonics
Source: Sci Rep. 2020 Jun 11;10:9461. doi: 10.1038/s41598-020-66324-y (PMC7289823; doi:10.1038/s41598-020-66324-y)
Supplement: Supplementary file 1 — Supplementary Information. [file 41598_2020_66324_MOESM1_ESM.pdf]

Supplementary information for:

Geochemical evidence for a widespread mantle re-enrichment 3.2 billion years ago: Implications for global-scale plate tectonics

Hamed Gamal EL Dien[1,2]*, Luc-Serge Doucet[1], J. Brendan Murphy[1, 3], Zheng-Xiang Li[1]

[1] Earth Dynamics Research Group, The Institute for Geoscience Research (TIGeR), School of Earth and Planetary Sciences, Curtin University, GPO Box U1987, Perth, WA 6845, Australia

[2] Geology Department, Faculty of Science, Tanta University, 31527 Tanta, Egypt

[3] Department of Earth Sciences, St. Francis Xavier University, Antigonish, Nova Scotia, Canada

[*] Corresponding author:

E-mail address: hamed.gamaleldien@postgrad.curtin.edu.au

Supplementary Figures

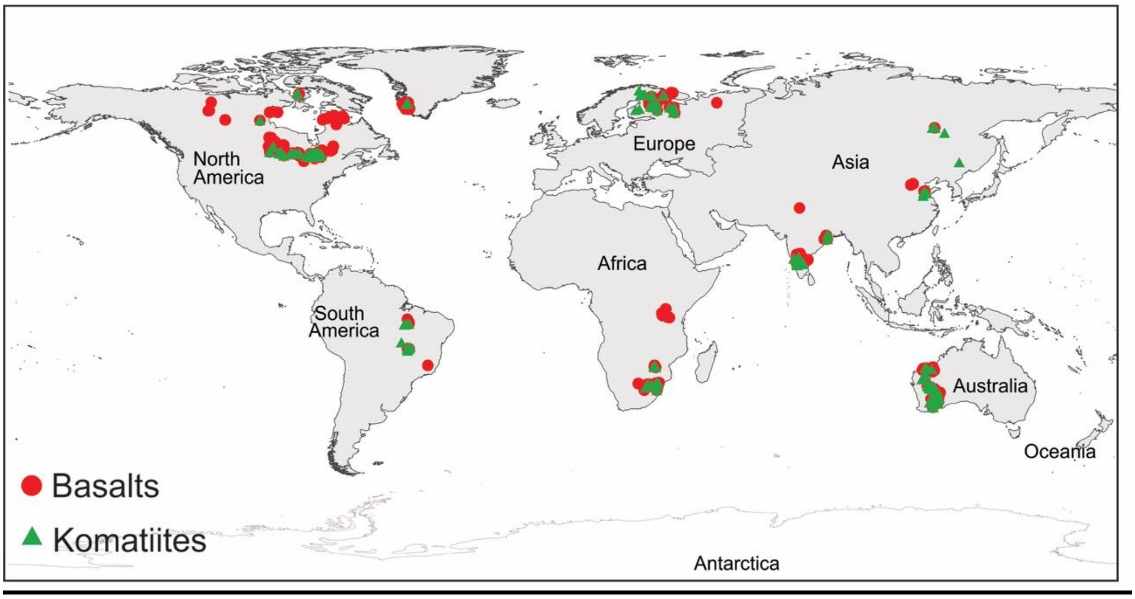

Supplementary Figure 1: Sample locations of basaltic rocks and komatiites used in this study. The map shows that the samples covered all the continents.This map created using ArcGIS Desktop 10.7 final-Curtin university licenced version (https://www.arcgis.com/home/index.html).

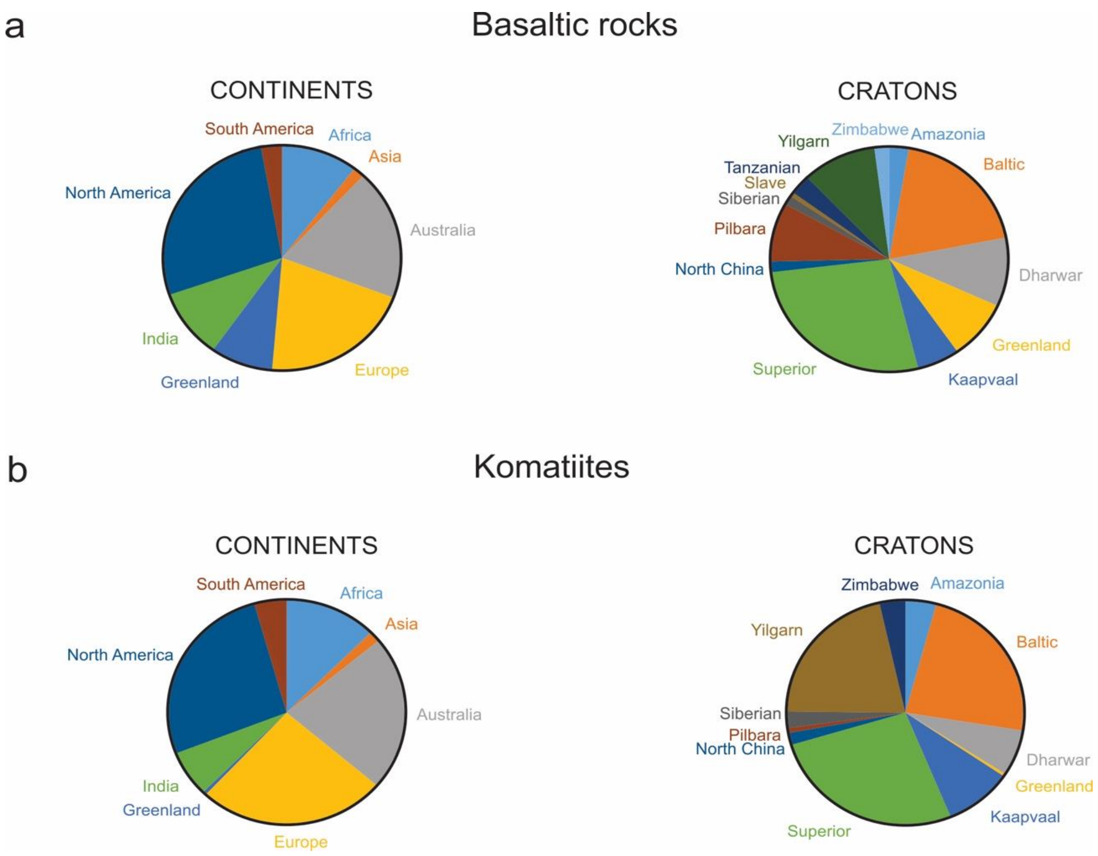

Supplementary Figure 2: 2D Pie-charts show that the distribution of the studied samples of basaltic rocks and komatiites is representative for all continents and cratons.

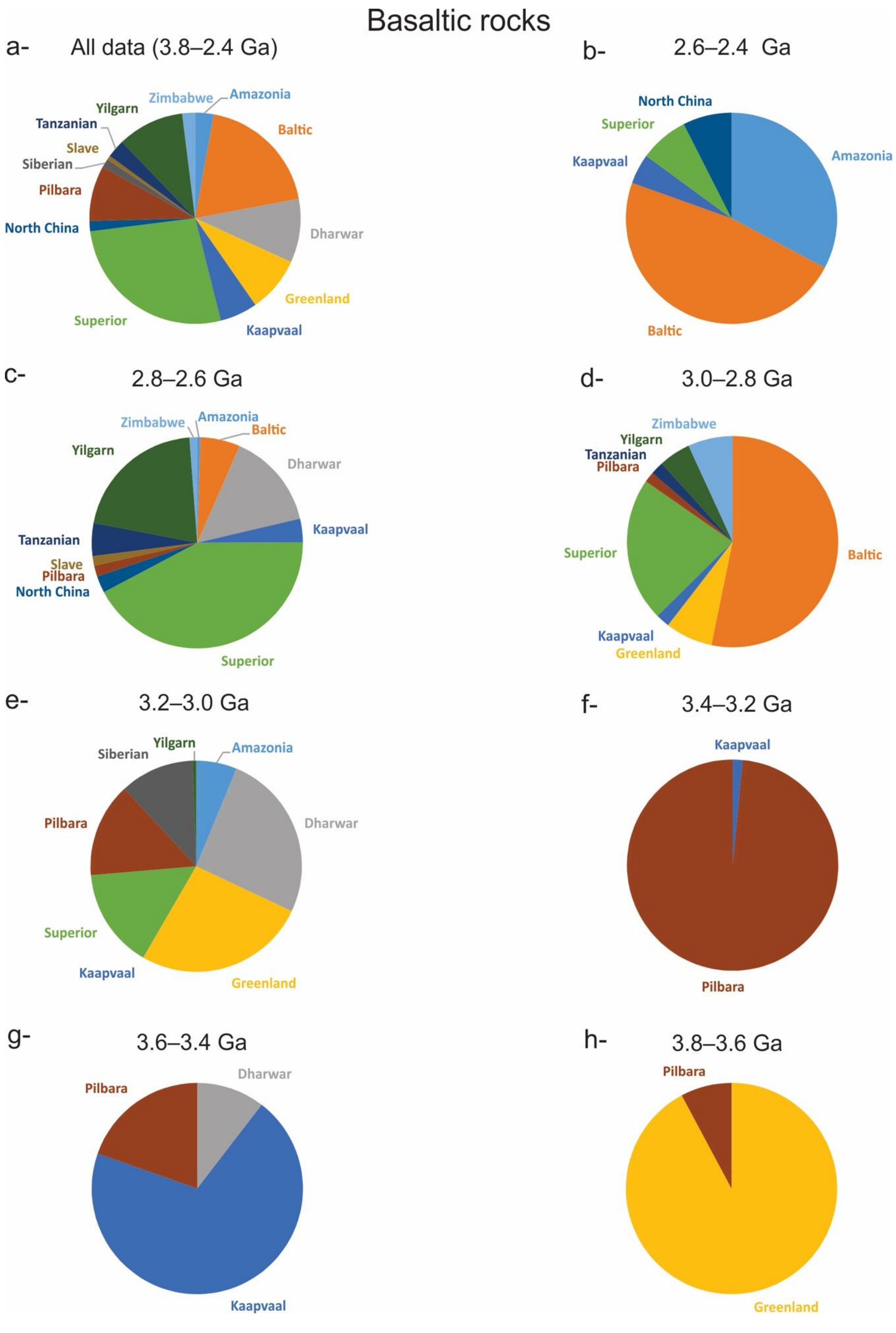

Supplementary Figure 3: **2D Pie-charts show that the distribution of the studied basalt samples is representative of all the cratons for the time range of 3.8–2.4 Ga.**

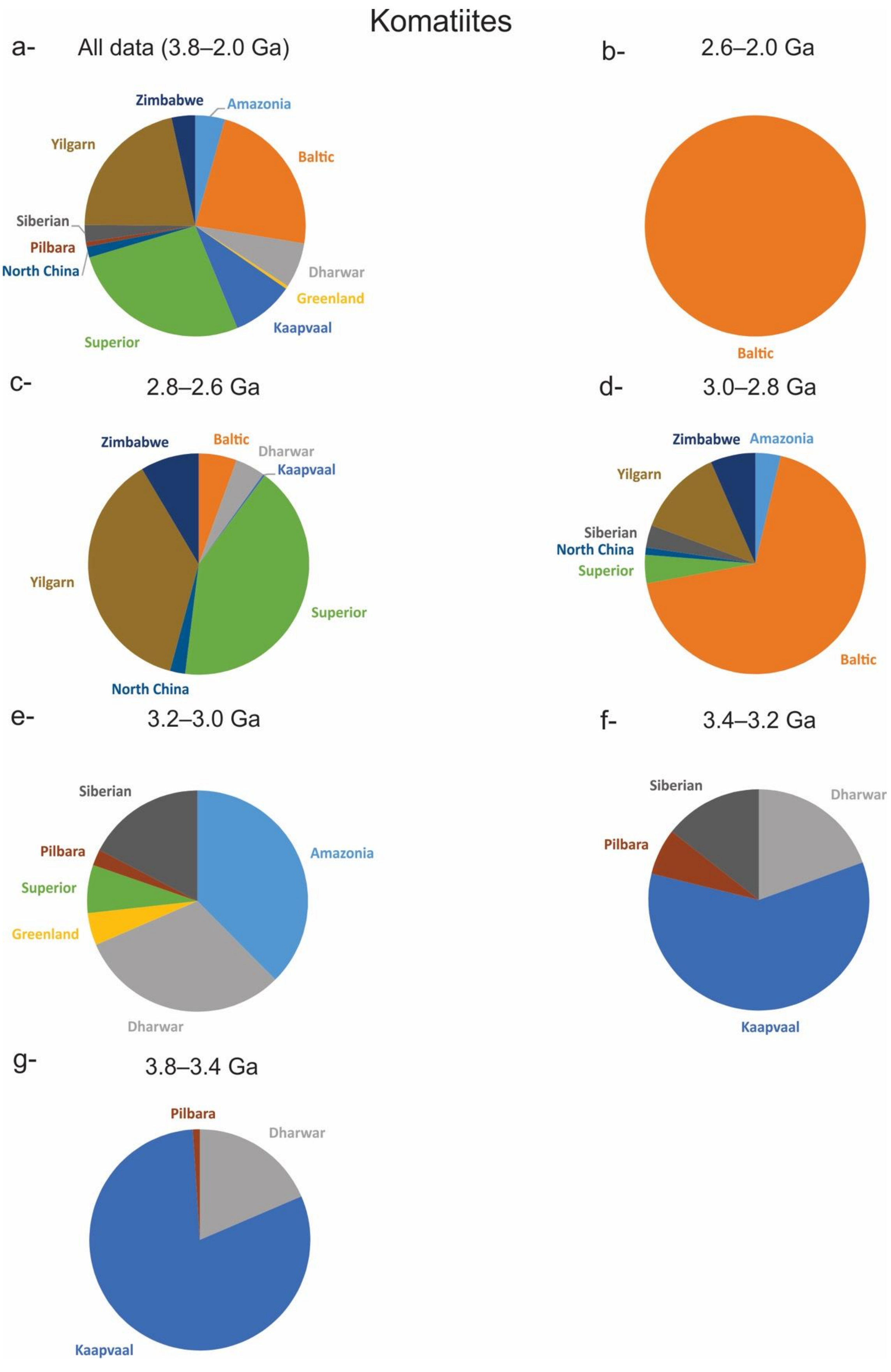

Supplementary Figure 4: **2D Pie-charts show that the distribution of the studied komatiite samples is representative of all the cratons for the time range of 3.8–2.0 Ga.**

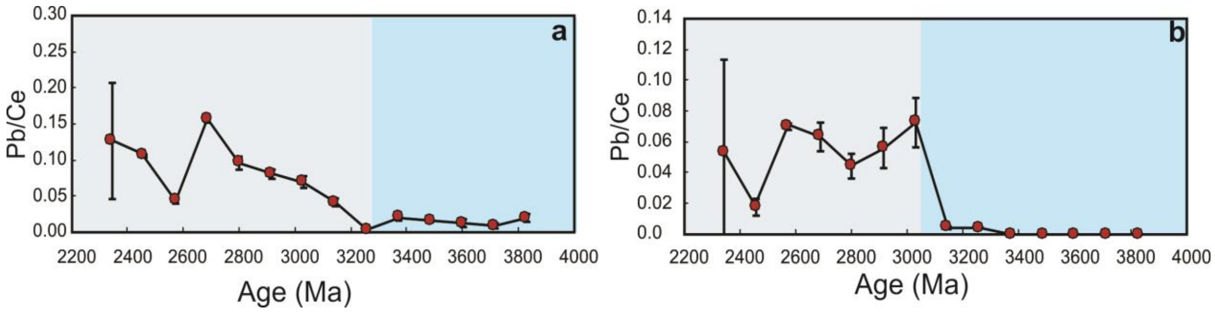

Supplementary Figure 5: **Time evolution of Pb/Ce ratios for basaltic rocks (a) and komatiites (b)**. Pb/Ce ratio shows an abrupt increase after ~3.25 Ga for basalts and after ~3.15 Ga for komatiites. Error bars show the 2-s.e.m. (standard error of the mean) uncertainties.

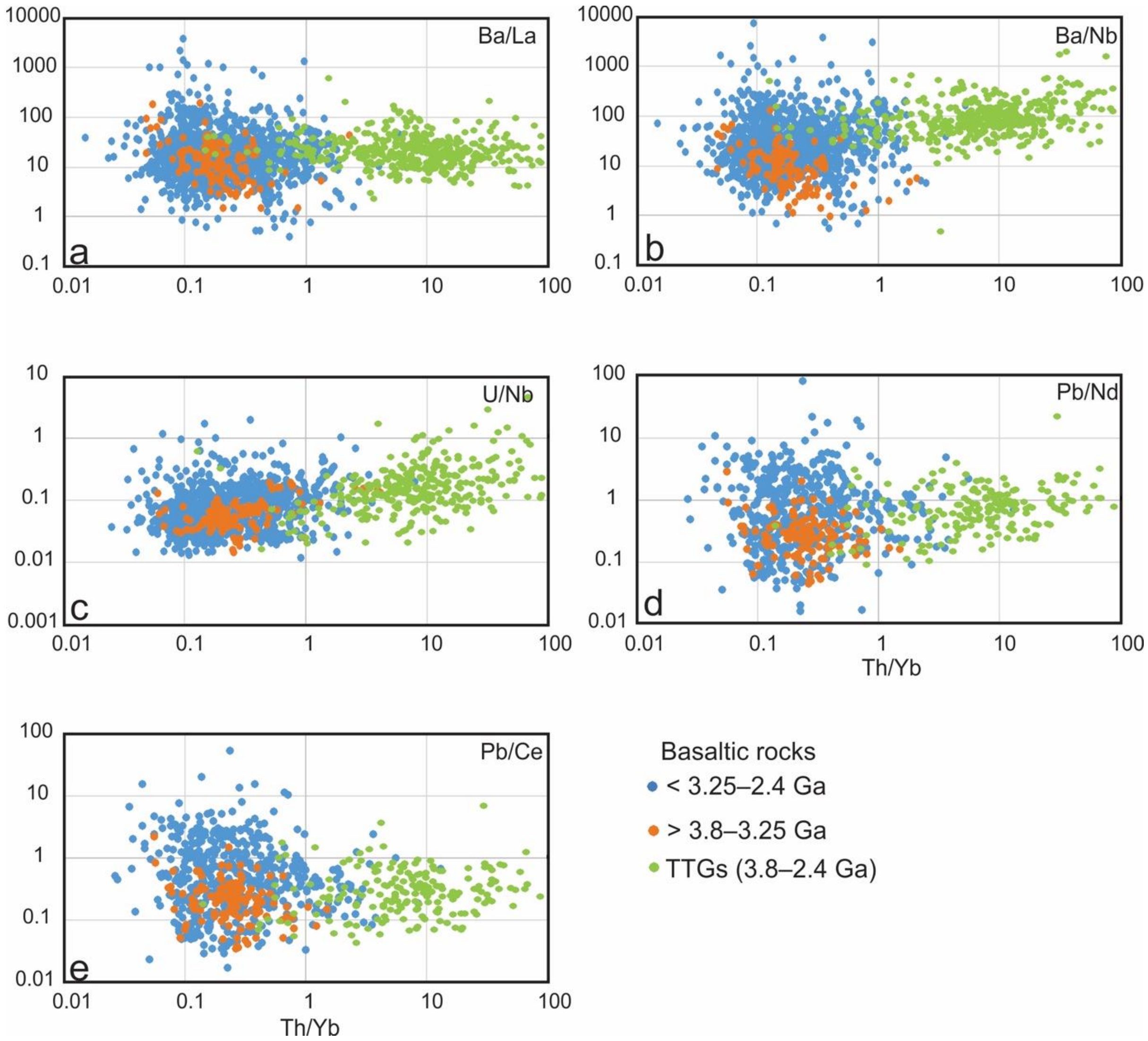

Supplementary Figure 6: Plots of Th/Yb vs Ba/La (a), Ba/Nb (b), U/Nb (c), Pb/Nd (d), and Pb/Ce (e) for basaltic rocks and TTGs. The basalts datasets are split into two age ranges <3.25–2.4 Ga and >3.8–3.25 Ga. Th/Yb is significantly higher for TTGs than for basalts, indicating no crustal contamination in the basalts.

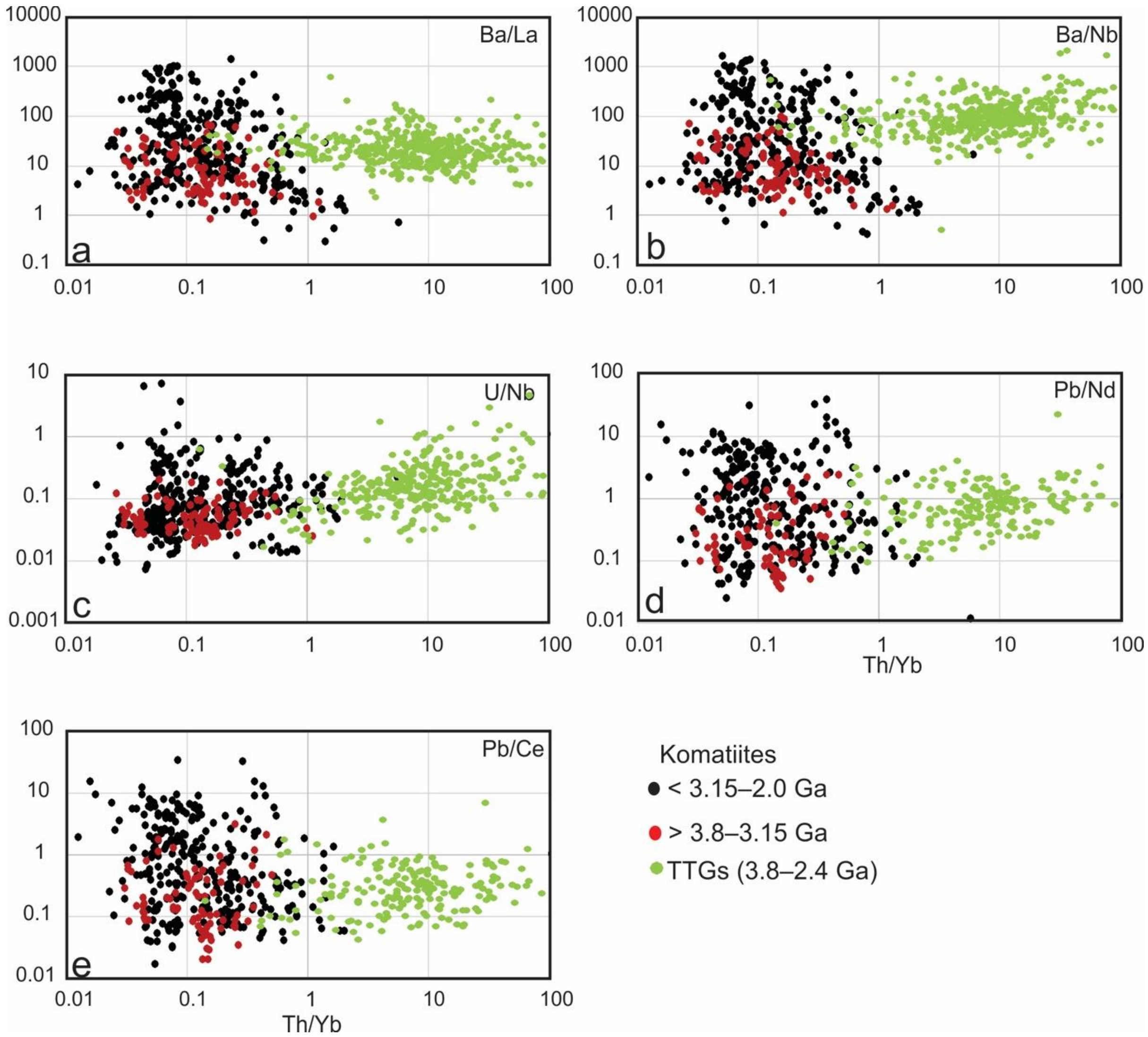

Supplementary Figure 7: **Plots of Th/Yb vs Ba/La (a), Ba/Nb (b), U/Nb (c), Pb/Nd (d), and Pb/Ce (e) for komatiites.** The datasets are split into two ranges as <3.15–2.0 and >3.8–3.15 Ga. Th/Yb is significantly higher in TTGs than in komatiites, indicating no crustal contamination in the komatiites.

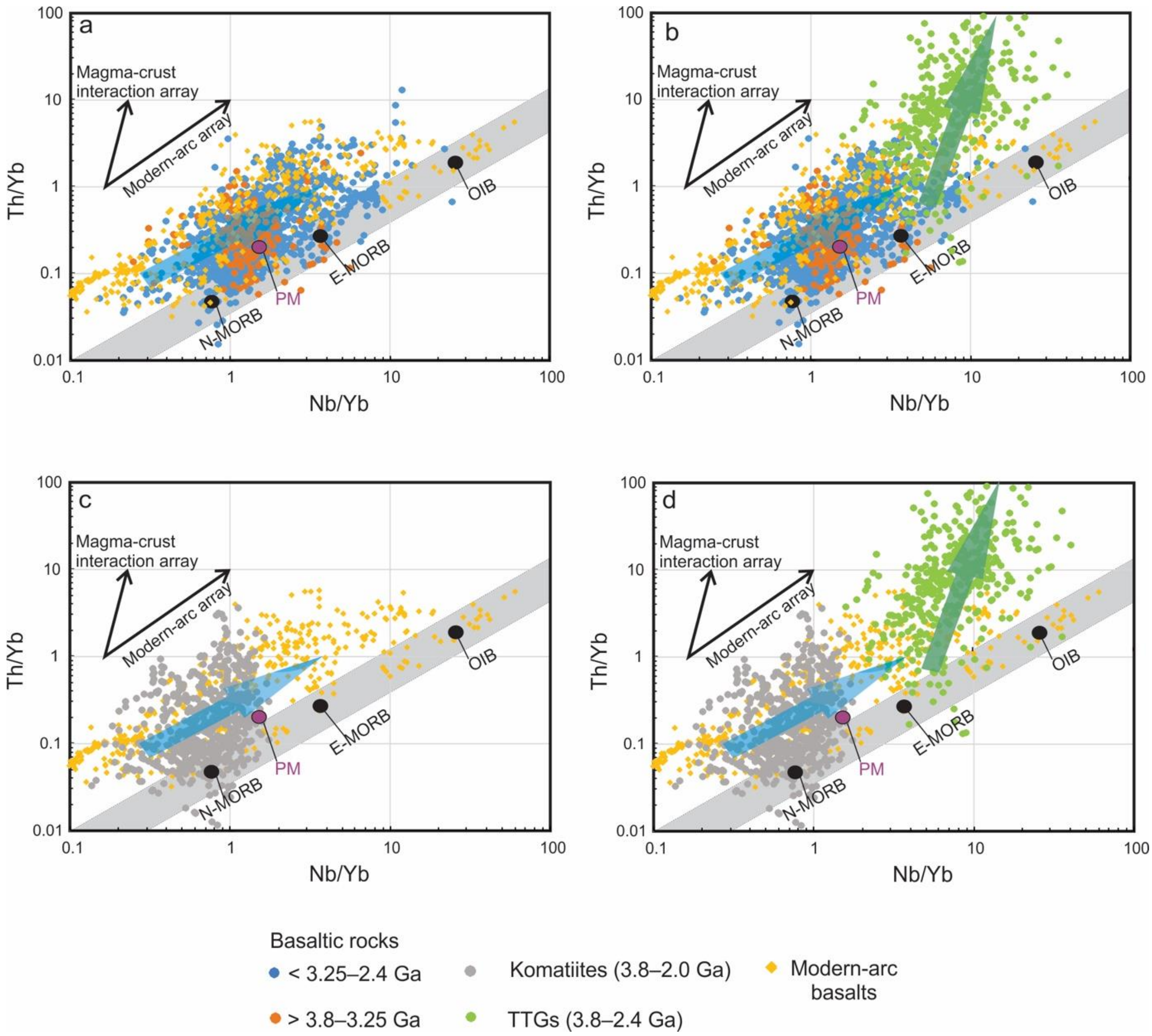

Supplementary Figure 8: **Nb/Yb-Th/Yb plots of Pearce[1] for the studied basalts (a, b), komatiites (c, d) and TTGs.** The primitive mantle value is from Ref[2], and the modern-arc basalts datasets are collected from Georoc. The basalt and komatiite datasets define a trend (bold blue arrows) parallel to the oceanic mantle array (grey field), as do modern arc-basalts. In contrast, TTGs have an oblique trend (bold green arrow). Moreover, the Th/Yb ratios of the TTGs are significantly higher than that of the basalts and komatiites, indicating no crustal contamination.

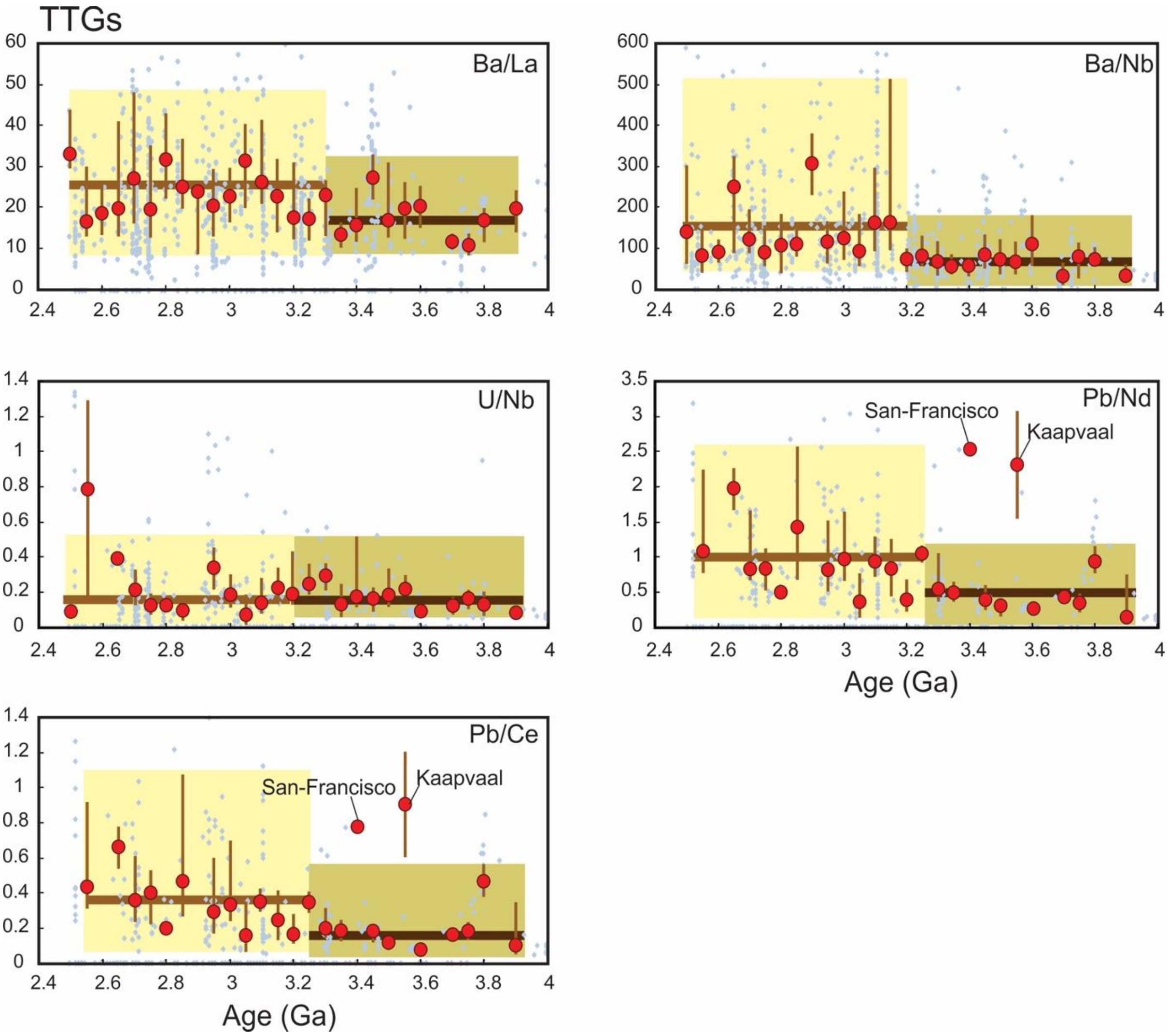

Supplementary Figure 9. **Plots of selected fluid-mobile/immobile elements ratios of tonalite-trondhjemite-granodiorite (TTG) rocks.** Ba/La (a), Ba/Nb (b), U/Nb (c), Pb/Nd (d), and Pb/Ce. Grey dots represent the entire data compiled from Ref[3]. Red dots represent the medians using 50 Myr windows, and vertical bars span across the middle 50% of the data, called here the median data range. The rectangular shades of different colours represent the median data range before and after ~3.3–3.2 Ga. Solid horizontal lines are the average of medians for data within each rectangular shade. For Pb/Nd and Pb/Ce, datasets from the Kaapvaal and San-Francisco cratons show abnormal increase at 3.55 and 3.4 Ga which we take as representing local events. All but the U/Nb ratios show a step change to a higher value after ~3.3–3.2 Ga.

Refreneces

1. Pearce, J. A. Geochemical fingerprinting of oceanic basalts with applications to ophiolite classification and the search for Archean oceanic crust. *Lithos* **100**, 14–48 (2008).
2. McDonough, W. . & Sun, S. –. The composition of the Earth. *Chem. Geol.* **120**, 223–252 (1995).
3. Johnson, T. E. *et al.* Secular change in TTG compositions: Implications for the evolution of Archaean geodynamics. *Earth Planet. Sci. Lett.* **505**, 65–75 (2019).
